# Dietary Valine Requirement of Juvenile Olive Flounder (*Paralichthys olivaceus*)

**DOI:** 10.1155/2024/3643845

**Published:** 2024-07-25

**Authors:** Jaehyeong Shin, Deahyun Ko, Mirasha Hasanthi, Gunho Eom, Kyeong–Jun Lee

**Affiliations:** ^1^ Department of Marine Life Sciences Jeju National University, Jeju 63243, Republic of Korea; ^2^ Marine Life Research Institute Kidang Marine Science Institute Jeju National University, Jeju 63333, Republic of Korea

## Abstract

This study was conducted to estimate dietary valine (Val) requirement for juvenile olive flounder (*Paralichthys olivaceus*). In a feeding trial, a total of 540 fish (initial body weight: 23.0 ± 0.2 g) were stocked into 18 tanks (210 L). Six experimental diets containing graded levels of Val (4, 8, 12, 16, 20, and 24 g/kg, dry matter basis) were fed to the fish in triplicate groups for 13 weeks. The lowest growth, feed utilization, and survival were observed in 4 g/kg Val group (*P* < 0.05). Dietary Val deficiency resulted in significant decreases (*P* < 0.05) in whole-body protein and Val concentrations, hepatosomatic index, condition factor and plasma protein and cholesterol levels. Nonspecific immunity and antioxidant activities were significantly lower (*P* < 0.05) in 4 g/kg Val group than in other groups. Dietary Val deficiency upregulated the expression of proinflammatory cytokines and downregulated the expression of anti-inflammatory cytokines and intestinal tight junction protein (*occludin*) (*P* < 0.05). Mucosal fold height and submucosa and muscularis thickness of fish intestine were significantly lower (*P* < 0.05) in fish fed 4 g/kg Val diet. Relatively lower lipid droplet in hepatic cell was observed in 4 g/kg Val group. Our findings suggested that dietary Val requirements for juvenile olive flounder would be 17.7–18.9 g Val/kg (35.4–37.8 g/kg on the basis of crude protein), estimated by quadratic regression analysis based on the weight gain, protein efficiency ratio, and protein retention efficiency.

## 1. Introduction

Valine (Val) is an essential amino acid (EAA) in fish feeds and plays important roles in the synthesis of protein and amine neurotransmitters, maintenance of nitrogen balance, and tissue repair [1]. Along with isoleucine (Ile) and leucine (Leu), Val belongs to the group of branched chain amino acid (BCAA), which are major components of globular proteins and plays important roles in maintaining cell structure and the creation of body protein and muscle [2]. In addition, previous studies reported that BCAA contribute to lipolysis, glucose metabolism, intestinal development, and immune responses in mice [3, 4]. In weanling piglets, dietary BCAA has been reported to enhanced intestinal expression of amino acid (AA) transporters, intestinal development, and growth performance [5]. Dietary Val deficiency was reported to induce impaired digestion and growth and decreased innate immunity and antioxidant capacity in fishes, such as mrigal carp (*Cirrhinus mrigala*) [6], red seabream (*Pagrus major*) [7], and grass carp (*Ctenopharyngodon idella*) [8]. Therefore, determination of dietary Val requirements is essential to achieve maximum growth, nonspecific immune response, and antioxidant capacity in the aquafeed industry.

The intestine is one of the most important immune organs in fishes. The intestinal immune system of fish prevents the infection of various pathogenic organisms and protects the intestinal cells from toxic compounds [9]. Luo et al. [8] described that dietary Val levels are closely related to intestinal immune response regulation and intestinal morphological development of fishes. Impaired intestinal immune system can be resulted to pathogenic infections, inflammation, and deformation of the intestinal structure [10]. Although, dietary Val levels have suggested to important for optimum growth and immune responses in fish, few studies have assessed the interaction between intestinal immune status and dietary Val levels.

Olive flounder (*Paralichthys olivaceus*) is the most important fish species in South Korean aquaculture, and its annual production reached 45,801 tons in 2022, accounting for approximately 51% of total annual finfish production [11]. Nonetheless, to the best of our knowledge, the dietary Val requirements has not been yet determined for olive flounder. Therefore, this study sought to estimate the optimal dietary Val level for juvenile olive flounder based on growth and protein efficiency, and evaluate the effects of dietary Val on immune responses, gene expression of tight junction (TJ) proteins, and inflammatory cytokines and intestinal.

## 2. Materials and Methods

### 2.1. Animal Ethics Statement

This work was reviewed and approved by the Guidelines of the Animal Care and Use Committee of Jeju National university (2019–0031).

### 2.2. Experimental Diets

The basal diet was formulated considering protein, energy, and essential AAs requirements of olive flounder [12, 13]. Analyzed the chemical composition of ingredients and prepared diet formulations. Six isonitrogenous (50% crude protein) and isocaloric (18.5 MJ/kg) semipurified diets were formulated with crystalline AA mixture (excluding Val), and sardine fish meal (Orizon S.A., Santiago, Chile) was used as the main protein sources for a basal diet (Table 1). The energy value of diet was estimated on the basis of physiological fuel value, i.e., 3.43 kcal/g proteins or carbohydrates and 0.99 kcal/g lipids [14]. Crystalline Val (purity ≥ 99%, Shanghai Ajinomoto, Shanghai, China) was added at the expense of L-alanine (purity ≥ 99%, Vixxol, Gyeonggi, South Korea) to the basal diets with graded levels of 0, 4, 8, 12, 16, and 20 g/kg to contain target Val levels of 4, 8, 12, 16, 20, and 24 g/kg diet, respectively. The analyzed Val concentration in the semipurified diets was 5.0, 8.6, 12.3, 15.7, 18.9, and 23.5 g/kg (Table 2). All the ingredients, cod liver oil, and 10% water were mixed using a mixer and pelletized by 3 mm mash using a feed maker (SP-50, Kumkang Engineering, Daegu, South Korea). The experimental diets were dried at 20°C in dry oven for 15 hr and stored at −24°C until use.

### 2.3. Fish and Feeding Trial

Olive flounder juveniles were purchased from Yeongyeong farm (Jeju, South Korea), and the experiment was conducted in the Institute of Marine Sciences of Jeju National University (Jeju, South Korea). Olive flounder juveniles were acclimatized to the experimental condition for 12 days before feeding trial. A total of 540 fish (average body weight: 23.0 ± 0.2 g) were stocked into eighteen 210-L polypropylene tanks (30 fish per tank) in triplicates per dietary group. The fish were fed three times daily (08:30, 13:30, and 19:00 hr) until they were apparently satiated for 13 weeks feeding trial. The rearing water was regulated to a flow rate of 3 L/min/tank with filtered seawater, and aeration was installed to maintain dissolved oxygen. The photoperiod was maintained using fluorescent lights to maintain 12 hr light:12 hr dark. Water temperature and dissolved oxygen were measured using a Pro20 Dissolved Oxygen Meter (YSI, Yellow springs, OH, USA), salinity was measured using a Master-S28M Salinity Refractometer (ATAGO, Tokyo, Japan), pH was measured using a SevenCompact pH meter S210 (Mettler Toledo, Columbus, OH, USA), and ammonia nitrogen level was analyzed by the Strickland and Parsons [15] method. Water conditions in all experimental tanks were maintained during feeding trial as follows: water temperature of 26.1 ± 2.1°C, dissolved oxygen of 7.5 ± 1.2 mg/L, salinity of 31.0 ± 0.4 g/L, pH of 7.0 ± 0.5, and total ammonia nitrogen of 0.012 ± 0.001 mg/L. Water conditions did not negatively affect the olive flounder [16, 17, 18].

### 2.4. Sampling Procedure

After the 13 weeks feeding trial, all the fish in each tank was individually counted and weighed to calculate specific growth rate (SGR), weight gain (WG), feed intake (FI), protein efficiency ratio (PER), protein retention efficiency (PRE), feed conversion ratio (FCR), feed efficiency (FE), and survival. Fish length was measured to calculate condition factor (CF). Six fish from each tank were randomly collected and anesthetized by 120 mg/L 2-phenoxyethanol solution (Sigma–Aldrich, St. Louis, MO, USA). Then, fishes were dissected to collect the intestine, liver, and stomach. The collected organs were weighed to calculate the intestinesomatic index (ISI), stomachsomatic index (SSI), and hepatosomatic index (HSI). The intestine samples were stored in Bouin's solution for histological analysis and in liquid nitrogen for RNA extraction. Blood samples were collected from the caudal veins of three fish in each tank by using heparinized syringes for plasma and nonheparinized syringes for serum. Serum samples were kept under room temperature for 20 min for clotting. Then, samples were centrifuged at 5,000 x *g* for 10 min to separate serum from clotted samples and plasma from heparinized samples. Both were then stored at −80°C until used for analysis.

### 2.5. Analytical Methods

Moisture (125°C, 3 hr) and ash (550°C, 4 hr) levels in the experimental diets and whole body were analyzed using standard procedures [19]. Protein and lipid were measured using Kjeltec Analyzer Unit 2300 (Kjeltec™ 2300, FOSS analysis, Hillerød, Denmark) and Soxhlet extraction method (Soxhlet Extraction System C-SH6, South Korea), respectively. The AA profiles were analyzed by ninhydrin method [20] using an automatic AA analyzer (S433, Sykam GmbH, Fürstenfeldbruck, Germany). Hematocrit analysis was performed by a microhematocrit equipment (VS-12000, Vision Scientific, Daejeon, South Korea). Plasma alanine aminotransferase (ALT), aspartate aminotransferase (AST), total protein, cholesterol, and glucose levels were determined using an automatic biochemistry analyzer (SLIM, SEAC Inc, Florence, Italy). Nitro blue tetrazolium (NBT) activity in whole blood was assayed as the method described by Anderson and Siwicki [21]. Immunoglobulin (Ig), lysozyme, myeloperoxidase (MPO), and antiprotease activities in plasma and serum were assayed as the methods described by Siwicki et al. [22], Hultmark et al. [23], Quade and Roth [24], and Ellis [25], respectively. The level of glutathione peroxidase (GPx) and superoxide dismutase (SOD) in plasma was detected by commercial kits with code of K762 and K335 (Biovision, Inc., Milpitas, CA, USA), respectively.

The intestines were sampled from three fish per tank and homogenized by a tissue grinder (Kimble Chase, Vineland, New Jersey, USA), which was followed by RNA extraction from the tissue suspension by using an RNA extraction kit (Takara, Japan). The quantity and quality of whole RNA were measured using a NanoDrop 2000 (Thermo Scientific, Wilmington DE, USA). The 260/280 nm ratios of all samples ranged from 1.85 to 1.96. cDNA synthesis was performed using PrimeScript™ 1st strand cDNA synthesis kit (Takara, Japan). The primer sequences and information are shown in Table 3. Analysis of gene expression was performed by a thermal cycler dice (real-time system Ⅲ, Takara, Japan) using TB Green Premix Ex Taq kit (Takara, Japan). The reaction was performed in a 10 *μ*L sample containing 5 *μ*L of TB Green Master Mix, 1.2 *μ*L RNase-free dH_2_O, 3 *μ*L cDNA template, and 0.4 *μ*L each pair of primer. Cycling conditions were 95°C for 2 min followed by 40 cycles of 95°C for 10 s, 59°C for 15 s, and 72°C for 10 s. The results of gene expression analysis were evaluated using the 2^−*ΔΔ*CT^ method and normalized to *β*-actin.

The intestine samples in Bouin's solution were dehydrated using a tissue processor (TP1020, Leica. Wetzlar, Germany). After dehydration, samples were embedded in paraffin, sectioned at 6 *μ*m thickness, placed in glass slides, and stained with hematoxylin and eosin. The stained slides were observed using an Olympus CKX41 (Tokyo, Japan) microscope equipped digital camera (DIXI Optics, Daejeon, South Korea). Mucosal fold height and width, lamina propria thickness, submucosal thickness, and muscularis thickness were measured using the image analysis software ImageJ 1.32j (National Institutes of Health, Bethesda, MD, USA).

### 2.6. Statistical Analyses

All data were expressed as mean ± standard deviation. Data were analyzed using one-way analysis of variance (ANOVA). All the data were statistically analyzed using SPSS 24.0 (SPSS Inc., Chicago, IL, USA). Tukey's post hoc test was used to compare the mean values when ANOVA revealed differences between the groups. The percentage data were transformed into arcsine values before analyses. Statistical differences in data were considered as significant at *P* < 0.05. An examination of orthogonal polynomial contrasts was conducted to ascertain the linear and/or quadratic nature of the effect. Optimal dietary Val requirements were quantified by quadratic regression analysis, following the method recommended by Parr et al. [27].

## 3. Results

### 3.1. Growth Performance and Feed Utilization

**Figure 1:**
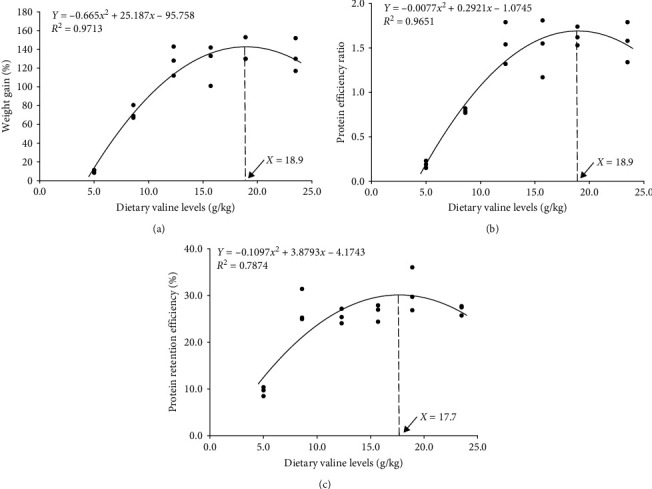
Quadratic regressions of the relationship between dietary inclusion valine levels and growth performance of juvenile olive flounder (*P. olivaceus*). (a) The regression analysis between weight gain (%) and dietary valine level; (b) the regression analysis between protein efficiency ratio and dietary valine level; (c) the regression analysis between protein retention efficiency (%) and dietary valine level.

Fish growth (final body weight (FBW), WG, and SGR) was significantly enhance (*P* < 0.05) with an increase in dietary Val level up to 12 g/kg diet (Table 4). FCR was significantly increased (*P* < 0.05) in 4 g/kg Val group than in other groups. FE and PER were significantly improved (*P* < 0.05) in 12, 16, 20, and 24 g/kg Val groups, whereas 4 g/kg Val group showed the lowest. PRE was significantly lower (*P* < 0.05) in 4 g/kg Val group than in other groups. However, FI was not significantly affected (*P* > 0.05) by different levels of dietary Val. Survival was significantly lower (*P* < 0.05) in 4 g/kg Val group compared to that of fish fed other diets containing higher Val levels. On the basis of quadratic regression, the dietary Val requirements of juvenile olive flounder (23–54 g), based on WG, PER, and PRE, were estimated to be 17.7–18.9 g/kg (35.4–37.8 g/kg on the basis of crude protein) (Figure 1). FBW, WG, SGR, FCR, FE, PER, PRE, and survival had significant linear and quadratic trends (*P* < 0.05) by the dietary Val levels.

HSI was significantly lower (*P* < 0.05) in 4 and 8 g/kg Val groups than 12 g/kg Val group (Table 5). SSI was not significantly affected (*P* > 0.05) by the dietary Val levels. ISI was significantly reduced (*P* < 0.05) in 20 and 24 g/kg Val groups than in fish fed 4 and 8 g/kg Val diets. CF was significantly lower (*P* < 0.05) in 4 g/kg Val group compared to that of 12, 16, 20, and 24 g/kg Val groups. HSI and CF results showed significant linear and quadratic trends (*P* < 0.05), while ISI showed significantly linear trends.

### 3.2. Whole-Body Proximate Composition and AA Profile

Whole-body protein level of the fish was significantly lower (*P* < 0.05) in 4 g/kg Val group than in other groups (Table 6). Whole-body ash level was significantly higher (*P* < 0.05) in 8, 12, 16, 20, and 24 g/kg Val groups compared to that of fish fed 4 g/kg Val diet. In contrast, moisture level was significantly higher (*P* < 0.05) in fish fed the basal diet containing 4 g/kg Val than in fish fed other diets. Whole-body lipid level was not significantly affected (*P* > 0.05) by the dietary Val levels. Whole-body protein and moisture levels exhibited significant linear and quadratic trends (*P* < 0.05), while ash level showed significant quadratic trends (*P* < 0.05) to the dietary Val levels.

In the results of whole-body AA concentrations, Val (linear, quadratic; *P* < 0.05), Ile (linear, *P* < 0.05), Leu (linear, *P* < 0.05), threonine (linear, *P* < 0.05), and serine (linear, *P* < 0.05) showed significant increase with the increasing dietary Val levels (Table 7). Other AA levels were not significantly affected (*P* > 0.05) by the dietary Val concentrations.

### 3.3. Hematological Parameters, Nonspecific Immunity, and Antioxidant Capacity

Total protein and cholesterol concentrations in the plasma were significantly lower (*P* < 0.05) in 4 g/kg Val group than in other groups (Table 8). Hematocrit and ALT levels were not significantly affected (*P* > 0.05) by the dietary Val levels. Glucose level was significantly lower (*P* < 0.05) in 16, 20, and 24 g/kg Val groups compared to that of 4 g/kg Val group. AST level was decreased with an increase in dietary Val levels up to 24 g/kg. Fish groups fed 20 and 24 g/kg Val diets showed significantly lower (*P* < 0.05) AST values than other groups. Cholesterol, total protein, glucose, and AST showed significant linear and quadratic trends (*P* < 0.05).

NBT activity was significantly lower (*P* < 0.05) in fish fed 4 g/kg Val diet in comparison with other dietary groups (Table 9). MPO activity was significantly improved (*P* < 0.05) in 16 and 24 g/kg Val groups than in fish fed 4 g/kg Val diet. Ig level was significantly increased (*P* < 0.05) in 24 g/kg Val group in comparison with the basal group. SOD activity was significantly higher (*P* < 0.05) in 8, 12, 16, and 24 g/kg Val groups than in 4 g/kg Val group. GPx activity was significantly improved (*P* < 0.05) in fish fed 12, 16, and 20 g/kg Val diets than in 4 g/kg Val group. Lysozyme and antiprotease activities did not show any significant differences (*P* > 0.05) among the dietary groups. NBT, SOD, and GPx had significant linear and quadratic trends (*P* < 0.05), while MPO and Ig showed significant linear trends (*P* < 0.05).

### 3.4. Intestinal Gene Expression

**Figure 2:**
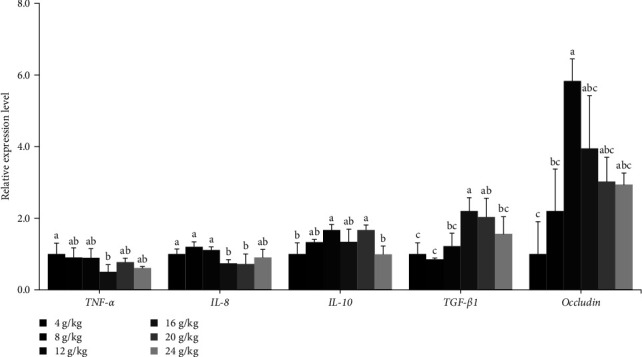
Relative gene expression of tumor necrosis factor-*α* (*TNF-α*), interleukin-8 (*IL-8*), interleukin-10 (*IL-10*), transforming growth factor-*β*1 (*TGF-β1*), and *occludin* in the intestine of olive flounder (*P. olivaceus*) fed the experimental diets. The experimental diets were formulated to contain valine by 4, 8, 12, 16, 20, and 24 g/kg diet. Gene expression were normalized to *β-actin* and expressed relative to control. Bars with different letters are significantly different (*P* < 0.05).

Interleukin-8 (*IL-8*) expression was significantly enhanced (*P* < 0.05) in fish fed diets containing 4–12 g/kg Val than in 16 and 20 g/kg Val groups (Figure 2). The lowest expression of tumor necrosis factor-*α* (*TNF-α*) was observed in 16 g/kg Val group (*P* < 0.05). Among anti-inflammatory cytokines, interleukin-10 (*IL-10*) expression was significantly upregulated (*P* < 0.05) in 12 and 20 g/kg Val groups than in fish fed 4 and 24 g/kg Val diets. Transforming growth factor-*β*1 (*TGF-β1*) gene expression was significantly upregulated (*P* < 0.05) in 16 and 20 g/kg Val groups compared to that of 4 and 8 g/kg Val groups. In the gene expression analyses related to TJ proteins, *occludin* expression was significantly increased (*P* < 0.05) in 12 g/kg Val group than the 4 g/kg Val group.

### 3.5. Histomorphological Parameters

**Figure 3:**
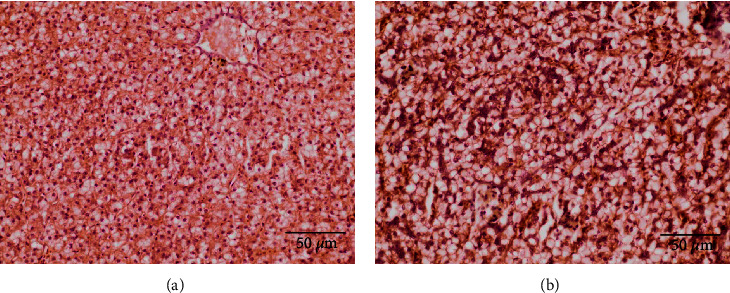
Hepatic lipid droplet accumulation in juvenile olive flounder (*P. olivaceus*) fed the 4 g/kg valine diet (a) and fed 12 g/kg valine diet. (b) Staining of hematoxylin and eosin.

**Figure 4:**
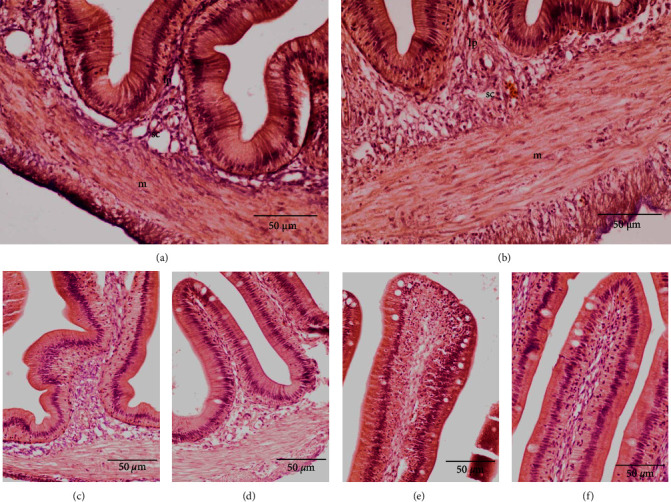
Distal intestine histology of juvenile olive flounder (*P. olivaceus*) fed 4 g/kg valine diet (a, c, and e) and 12 g/kg valine diet (b, d, and f). Fish fed 4 g/kg valine diet exhibits a shortening of mucosal folds and reduced submucosa and muscularis thickness, as well as a change in the outline of the mucosal fold. Lamina propria (lp), muscularis (m), submucosa (sc). Staining of hematoxylin and eosin.

In the histological analyses, the accumulation of lipid droplets in fish liver was relatively lower in fish fed a Val-deficient diet (4 g/kg) than in fish fed other diets (Figure 3). In the fish intestine, mucosal fold height was significantly increased (*P* < 0.05) in fish fed >8 g/kg Val diets (Table 10 and Figure 4). Submucosa and muscularis thickness were significantly increased (*P* < 0.05) in fish fed >8 g/kg Val diets than in fish fed the 4 g/kg Val diet. Mucosal fold and lamina propria thickness in intestine were not affected (*P* > 0.05) by the different Val levels. Mucosal fold height exhibited significant linear and quadratic trends (*P* < 0.05), while submucosa and muscularis thickness only showed significant linear trends (*P* < 0.05).

## 4. Discussion

In the present study, juvenile olive flounder showed low growth rates (18%–138%) in the feeding trial. Semipurified diets are recognized as having lower acceptability to fishes, as they are less palatable compared to practical diets resulting in less WG and feed efficiency. Olive flounder is a carnivorous fish and requires high protein levels (40%–60%) in the feeds [28]. Rahimnejad and Lee [7] found that red seabream showed very low growth rates (34%–154%) when they were fed a semipurified diet in a AA requirement study. Hernandez et al. [29] reported that olive flounder (1.59 g) showed poor WG (11.9–17.1 g) when they were fed a semipurified diets for 10 weeks. Thus, the low growth rate observed in the present study can be explained by the feed type of the fish.

A quadratic regression analysis based on WG, PER, and PRE estimated the optimal level of Val for juvenile olive flounder would be 17.7–18.9 g/kg in the diet. This level is similar to the reported requirements for golden pompano (*Trachinotus ovatus*), 19.9–20.2 g/kg in the diet [30]. However, it is higher than the requirements for red seabream, 9 g/kg [7]; mrigal carp, 15.2 g/kg [6]; grass carp, 14.0–14.5 g/kg [8]; Nile tilapia (*Oreochromis niloticus*), 11.5–12.7 g/kg [31]; Jian carp (*Cyprinus carpio*), 13.7 g/kg [32], blunt snout bream (*Megalobrama amblycephala*), 12.6–13.2 g/kg [33]; and hybrid grouper (*Epinephelus fuscoguttatus* ♀ × *E. lanceolatus* ♂), 15.6 g/kg [34]. The variations in AA requirement levels are attributed to the species, mathematical model, and growth stage [35].

Our findings from this study indicated that low dietary Val levels than 8 g/kg can induce poor growth, feed utilization efficiency, FI, and survival of juvenile olive flounder. Similar results were observed when a Val-deficient diet was fed to Nile tilapia [31], grass carp [8], and mrigal carp [6]. Val has been recognized as an important factor in protein synthesis because it is the main component of muscle in the fish body [2]. In addition, Val is essential for tissue growth and repair, controlling nitrogen balance and producing biological energy [1]. Many studies have reported that Val, as a BCAA, is closely associated with lipolysis, glucose metabolism, and intestinal development [3, 4]. Dietary Val deficiency has been reported to downregulate the target of rapamycin (TOR) in fish intestine [8] and gills [36]. The TOR signaling pathway is closely involved in energy consumption, thereby regulating protein synthesis, growth, and energy balance via hormonal signals [37]. TOR signaling is regulated by EAAs [38]. BCAAs are known to be regulators of protein synthesis and fish metabolism via TOR pathway [39]. TOR acts as an effective regulator in the brain to control FI and nutrient availability [40]. Unbalanced dietary EAA levels can disrupt blood EAA balance and thus inhibit activation of the general control nonderepressible 2 (GCN2) signaling pathway [41]. GCN2 detects a deficiency of AA and restricts the intake of diets that are lacking EAAs [42]. GCN2 activation can reduce protein translation from RNA [43]. GCN2 and TOR pathways play a central role in animals as major regulators controlling protein synthesis depending on AA levels in the blood [43]. Therefore, dietary Val deficiency could retard growth performance through metabolic disorders in fish. Several studies have indicated that excessive AA supplementation can lead to hyperaminoacidemia followed by retarded growth performance because the deamination of AAs is an energy-consuming process in fish [44]. Accordingly, excessive Val intake has been shown to reduce growth and feed utilization of Jian carp [32] and mrigal carp [6]. In contrast, the present study did not show any negative effect of excessive Val, even among fish that were fed high levels of Val (20–24 g/kg). Similar results have been reported in Nile tilapia [31] and blunt snout bream [33]. Thus, further study is recommended to estimate the effects of high dietary Val levels for olive flounder.

In this study, we found that dietary Val deficiency reduced the nonspecific immunity (NBT, MPO, and Ig) and antioxidant capacity (SOD and GPx) of the fish suggesting that dietary Val levels are directly involved in the immune status of olive flounder. Similarly, Rahimnejad and Lee [7] reported that dietary Val deficiency reduced the NBT, MPO activity, and Ig level in red seabream. NBT activity is usually used to quantify superoxide anion production in leucocytes to assess fish health [45]. MPO is an enzyme released by neutrophils that helps kill bacteria by making reactive oxygen molecules [46]. Immunoglobulins, also known as antibodies, are glycoproteins that play a key role in the fish immune response by recognizing and neutralizing harmful pathogens [46]. BCAAs provide the *α*-amino group for glutamine synthesis [47]. Glutamine is generally used for cytokine and antibody synthesis, macrophage activation, apoptosis inhibition, and regulation of T-lymphocyte proliferation [48]. In an *in vitro* test, Val deficiency completely suppressed the growth of lymphocytes [49]. It has been reported that lymphocytes are involved in the production of antibacterial compounds and are closely related to the immune responses of fish [50]. Li et al. [51] reported that BCAAs are directly involved in cytokine activation and antibody production through the TOR signaling pathway. Dietary Val deficiency downregulated TOR expression and disrupted immune responses of grass carp [8, 36], golden pompano [30], and red seabream [7]. SOD and GPX are important enzymes in cellular defense that maintain oxidative balance by counteracting superoxide radicals and peroxides [52]. Zhou et al. [34] reported that dietary Val deficiency leads to impairment of immune responses and antioxidant capacity in hybrid grouper. Therefore, the results in the present study indicate that dietary Val supplementation at an appropriate level is important to maintain normal immune responses of juvenile olive flounder.

The cholesterol concentration in fish blood was also affected by dietary Val levels in our study suggesting that low Val levels may disrupt cholesterol absorption, utilization, and metabolism. Cholesterol is known to be an essential component of cell membranes and also serves as a precursor to important metabolites such as bile acids and steroid hormones [53]. Fish can partially synthesize cholesterol by themselves, and acetyl-CoA is used for cholesterol synthesis from more than 20 enzymatic reactions steps [54, 55]. Rahimnejad and Lee [7] reported a significant decrease in cholesterol concentration in red seabream fed a Val-deficient diet. Similarly, the present results indicated that low concentrations of cholesterol in fish blood was resulted from Val deficiency. The low concentration of cholesterol could be due to the decreased absorption of dietary cholesterol in the intestinal lumen [56]. Histological changes in the intestines of fish fed the 4 g/kg Val diet might have affected the absorption of cholesterol in this study.

Hematological parameters are used to determine the overall health status of fish and identify chronic stress or metabolic disorders [57]. Xiao et al. [31] found that the blood components of fish were affected by dietary EAA levels and that plasma total protein concentration was enhanced by the dietary inclusion of Val. Similar results have been reported for red seabream [7]. AST and ALT play important roles in AA metabolism, and concentrations of these enzymes are generally used as an indicator of liver health status [58]. The levels of AST and ALT in plasma were affected by many stress factors such as lack of essential nutrients, ammonia toxicity, and water pollution [59]. Plasma glucose levels in fish are also increased by stress factors [60]. In the present study, the elevated levels of glucose, ALT, and AST in the blood of fish fed the basal diet which had the lowest Val level suggest that the dietary Val deficiency may cause a metabolic stress in olive flounder juveniles. Similar results have been reported for red seabream and Nile tilapia fed a Val-deficient diet [7, 31].

In this study, dietary Val deficiency downregulated intestinal anti-inflammatory cytokine (*TGF-β1* and *IL-10*) expression and upregulated proinflammatory cytokine (*TNF*-*α* and *IL*-*8*) expression in olive flounder juveniles. Inflammation is closely related to the intestinal immune status, and many cytokines are involved in the process of inflammation [10, 61]. *IL*-*8* and *TNF*-*α* are proinflammatory cytokines, and their upregulation is generally involved in inflammatory bowel disease [62]. In contrast, *TGF-β1* and *IL-10* are anti-inflammatory cytokines, and their expression is thought to counteract the production of proinflammatory cytokines [63]. Luo et al. [8] reported that Val deficiency upregulated *IL-8* and *TNF*-*α* and downregulated *IL-10* and *TGF-β1* in the intestine of grass carp. Fish growth is closely related to intestinal health, and the intestinal immune status is correlated with dietary nutrient levels [9]. Thus, our findings indicate that dietary Val deficiency has a negative effect on the intestinal health of olive flounder juveniles. On the other hand, *α*-melanocyte stimulating hormone (*α*-MSH) may be another factor underlying the interaction between dietary Val levels and intestinal inflammation [8]. *α*-MSH is produced by the pituitary gland and plays an important role in the regulation of skin color, feeding behavior, and energy metabolism of fish [64]. Val is a major component of *α*-MSH in fish [65]. Harris and Bird [66] showed that *α*-MSH stimulates anti-inflammatory cytokine production and inhibits proinflammatory effects in mammals. Therefore, in this study, dietary Val levels might have been partly related to the synthesis of *α*-MSH.

Our results showed that dietary Val deficiency significantly decreased intestinal *occludin* expression in the fish. Fish intestine has a physical barrier consisting of TJ complex and epithelial cells [67]. *Occludin* is known to be a component of TJ proteins in fish intestine [68]. Dietary Val deficiency has been reported to downregulate the gene expressions of proteins related to TJ complex (such as *occludin*, *zonula occludens-1*, and *claudin*) in grass carp intestine and gills [8, 36]. In the present study, the upregulated proinflammatory cytokine expression may have caused the downregulated *occludin* expression in the Val deficiency groups. Capaldo and Nusrat [69] found that the expression of *TNF-α* and *IL-8* was partly related to the regulation of TJ protein expression. Based on our results, dietary Val is likely to be involved in proinflammatory cytokine expression in fish intestine indicating that a reduced *occludin* expression may interact with increased expression of proinflammatory cytokines. However, the interaction between these two factors in fish is largely unknown.

In this study, we found that dietary Val deficiency caused histological changes in the intestine of juvenile olive flounder. The absorption ability of fish intestine is closely related to gut shape parameters such as mucosal fold height and width [70]. Dietary supplementation of Val was reported to increase the intestinal mucosal fold height of Jian carp [32]. Previous studies reported that AA deficiency in fish diets can impair the development of fish intestine [71]. The muscularis and submucosa play important roles in maintaining intestinal structure and function [72]. Macrophages in the muscularis are highly specialized cells that are essential for tissue homeostasis and protection of the intestine when injured or infected [73]. One important observation in this study was that dietary Val supplementation promoted the intestinal development of juvenile olive flounder.

Our results showed that the accumulation of hepatic lipid droplets was affected by dietary Val levels. Du et al. [74] reported that dietary Val deficiency caused significant changes in lipid metabolism in mice by reducing lipogenesis in the liver and increasing lipolysis in white adipose tissue. In addition, Solon-Biet et al. [4] found that dietary BCAA supplementation increased energy intake and hepatic fat accumulation in mice. These findings are consistent with our observations and suggest that Val-deficient diets, such as those tested in this study, may disrupt the energy metabolism of juvenile olive flounder. The HSI and SSI of juvenile olive flounder were significantly affected by dietary Val levels in this study. The development of digestive organs plays an important role in fish digestion and absorption ability. Dong et al. [32] reported that HSI and ISI of Jian carp were significantly increased in fish fed a diet containing sufficient levels of Val. However, only a few studies have reported the effect of dietary Val on the development of digestive organs. Thus, the present study results suggest that dietary Val deficiency may lead to an impairment in the development of fish digestive organs.

In this study, dietary Val deficiency decreased the whole-body protein level of juvenile olive flounder. This decrease may be attributable to a reduced protein synthesis caused by the dietary Val deficiency. This finding is also supported by lower PER, PRE, and FE and higher FCR in fish fed the Val-deficient basal diet. A lack of dietary EAA has been reported to reduce the whole-body protein level of fish [75]. Interestingly, whole-body concentrations of BCAAs (Val, Ile, and Leu) were also significantly lower in fish fed the basal diet. BCAAs are known to be absorbed in the intestine through a common transporter and two BCAA transaminase enzymes [76]. Harris et al. [77] reported that dietary BCAA deficiency or imbalance causes antagonistic effects in animals through the reduction of plasma BCAA concentration, which can reduce fish growth and feed utilization. This imbalance has been attributed to competitive inhibition during intestinal absorption and increased oxidation [78].

## 5. Conclusions

Dietary Val supplementation lower than 8 g/kg can delay the growth, hematological parameters, nonspecific immunity, and antioxidant capacity of juvenile olive flounder. In addition, dietary Val deficiency could upregulate the proinflammatory cytokines and downregulate the anti-inflammatory cytokines in the intestine of the fish. Our findings suggest that insufficient dietary Val supplementation could induce poor intestinal development in juvenile olive flounder. A quadratic regression analysis of WG, PER, and PRE indicates that the dietary Val requirements for juvenile olive flounder would be 17.7–18.9 g/kg (35.4–37.8 g/kg on the basis of crude protein).

## Figures and Tables

**Table 1:** Formulation of the experimental diets for juvenile olive flounder (*P. olivaceus*) (g/kg, dry matter basis).

Ingredients	Dietary valine levels (g/kg)					
	4	8	12	16	20	24
Fish meal, sardine^1^	100	100	100	100	100	100
AA mixture^2^	400	400	400	400	400	400
L-valine (99%)^3^	0	4	8	12	16	20
L-alanine (99%)^4^	20	16	12	8	4	0
Wheat flour^5^	100	100	100	100	100	100
Starch	200	200	200	200	200	200
Cod liver oil^6^	100	100	100	100	100	100
Taurine	10	10	10	10	10	10
Mineral mix^7^	20	20	20	20	20	20
Vitamin mix^8^	20	20	20	20	20	20
Guar gum^9^	20	20	20	20	20	20
Lecithin^10^	5	5	5	5	5	5
Choline	5	5	5	5	5	5
*Total*	1,000	1,000	1,000	1,000	1,000	1,000

^1^Orizon S.A., Corp., Santiago, Chile. ^2^Amino acids were by Vixxol Co., Ltd., Gyeonggi, South Korea. Amino acid mixture (g/kg) contains 66.8 g arginine, 26.7 g histidine, 44.9 g isoleucine, 83 g leucine, 108.6 g lysine, 29.5 g methionine, 44.4 g phenylalanine, 45.9 g threonine, 6.7 g tryptophan, 103.4 g aspartic acid, 151.5 g glutamic acid, 37.8 g serine, 32.6 g proline, 75 g glycine, 76.2 g alanine, 35.1 g tyrosine, and 31.9 g starch. ^3^Shanghai Ajinomoto Amino Acid Co. Ltd., Shanghai, China. ^4^Vixxol Co. Ltd., Gyeonggi, South Korea. ^5^Deahan Flour Co. Ltd., Incheon, South Korea. ^6^E- wha Oil & Fat Industry Corp., Busan, South Korea. ^7^Mineral mix was by AlphaAqua Co. Busan, South Korea. Mineral mix (g/kg) contains 80 g MgSO_4_·7H_2_O, 370 g NaH_2_PO_4_·2H_2_O, 130 g KCl, 40 g ferriccitrate, 20 g ZnSO_4_·7H_2_O, 356.5 g Ca-lactate, 0.2 g CuCl, 0.15 g AlCl_3_·6H_2_O, 0.01 g Na_2_Se_2_O_3_, 2 g MnSO_4_∙H_2_O, and 1.14 g CoCl_2_·6H_2_O. ^8^Vitamin mix was by AlphaAqua Co., Busan, South Korea. Vitamin mix (g/kg) contains 121.2 g L-ascorbic acid, 18.8 g DL-a tocopheryl acetate, 2.7 g thiamin hydrochloride, 9.1 g riboflavin, 1.8 g pyridoxine hydrochloride, 36.4 g niacin, 12.7 g Ca-D, pantothenate; 181.8 g myo-inositol, 0.27 g D, biotin; 0.68 g folic acid, 18.2 g p-aminobenzoic acid, 1.8 g menadione, 0.73 g retinyl acetate, 0.003 g cholecalciferol, 0.003 g cyanocobalamin, and 593.8 g starch. ^9^Vixxol Co. Ltd., Gyeonggi, South Korea. ^10^Lysoforte™ Dry, Kemin South Korea Co. Ltd., Seongnam, South Korea.

**Table 2:** Analyzed proximate composition and amino acid profile of the experimental diets (g/kg, dry matter basis).

Nutrient contents	Dietary valine levels (g/kg)					
	4	8	12	16	20	24
Proximate composition						
Crude protein	495	492	501	492	497	498
Crude lipid	109	105	102	104	105	107
Ash	32	31	31	31	32	31
Dry matter	953	953	954	951	952	956
Gross energy (MJ/kg)	18.5	18.5	18.5	18.5	18.5	18.5
Essential amino acids						
Valine	5.0	8.6	12.3	15.7	18.9	23.5
Histidine	14.4	14.4	13.8	14.1	13.5	13.9
Isoleucine	21.0	21.3	21.1	21.2	20.3	21.2
Leucine	38.1	39.1	38.6	38.6	36.5	38.5
Lysine	39.4	40.6	40.3	40.4	38.3	40.2
Phenylalanine	20.6	20.9	20.9	20.8	19.8	20.6
Threonine	20.5	21.0	19.7	20.6	19.3	20.8
Arginine	32.1	32.3	32.3	30.9	30.4	30.4
Nonessential amino acids						
Alanine	65.0	60.5	57.6	54.9	51.4	44.8
Aspartic acid	48.8	49.7	48.9	47.3	46.4	48.0
Glycine	35.3	34.2	34.8	33.7	35.4	34.7
Glutamic acid	76.4	77.8	76.9	75.6	76.1	76.1
Proline	20.5	20.5	21.8	22.2	19.3	19.6
Serine	17.1	17.2	16.6	16.2	15.4	15.7
Tyrosine	13.1	13.5	14.2	13.6	13.0	13.2
Total	467	472	471	466	454	461

**Table 3:** Primer sequences of gene expression for olive flounder (*P. olivaceus*).

Primers^1^	Sequences	Accession number/references
*TNF*-*α*		
Forward	5′–CGG CCA TCC ATT TAG AAG GTA GA–3′	Beck et al. [26]
Reverse	5′–GGG ATG ATG ATG TGG TTG TCC–3′	
*IL*-*8*		
Forward	5′–GCG ATA AAA CTC AGA TCA TTG CC–3′	Beck et al. [26]
Reverse	5′–TCT GAC CCC ATC TCT CGC CA–3′	
*IL-10*		
Forward	5′–TTT CAA AAG CCC GTT TGC GT–3′	KF025663.1
Reverse	5′–TTG GTT TCC TCC GTC ACT CC–3′	
*TGF-β1*		
Forward	5′–CAG CGA ACA CGA GCC AAA CAC–3′	Beck et al. [26]
Reverse	5′–TGT TCT GAG GGA TGG ACA TGG TG–3′	
*Occludin*		
Forward	5′–TCT TTG CTC TGA AGA CCC GC–3′	Beck et al. [26]
Reverse	5′–ATT GTT CAC CCA TGC CTC CA–3′	
*β-Actin*		
Forward	5′–TGC AGA AGG AGA TCA CAG CC–3′	HQ386788.1
Reverse	5′–ACT CCT GCT TGC TGA TCC AC–3′	

^1^ *TNF-α*, tumor necrosis factor-*α*; *IL-8*, interleukin-8; *IL-10*, interleukin-10; *TGF-β1*, transforming growth factor-*β*1.

**Table 4:** Growth performance, feed utilization, and survival of juvenile olive flounder (*P. olivaceus*) (initial body weight: 23.0 ± 0.2 g) fed the experimental diets for 13 weeks.

Item	Dietary valine levels (g/kg)						Pr > *F* ^*∗*^		
	4	8	12	16	20	24	ANOVA	Linear	Quadratic
FBW^1^	25.5 ± 2.99^c^	39.2 ± 1.76^b^	52.7 ± 4.03^a^	51.9 ± 5.16^a^	54.8 ± 4.01^a^	53.1 ± 4.20^a^	<0.001	<0.001	<0.001
WG^2^	10.1 ± 1.61^c^	72.2 ± 7.33^b^	127 ± 15.5^a^	125 ± 21.7^a^	138 ± 13.2^a^	133 ± 18.1^a^	<0.001	<0.001	<0.001
SGR^3^	0.18 ± 0.16^c^	0.61 ± 0.05^b^	0.92 ± 0.08^a^	0.91 ± 0.11^a^	0.97 ± 0.06^a^	0.95 ± 0.09^a^	<0.001	<0.001	<0.001
FCR^4^	14.7 ± 2.14^a^	2.47 ± 0.09^b^	1.28 ± 0.20^b^	1.34 ± 0.31^b^	1.21 ± 0.08^b^	1.27 ± 0.18^b^	<0.001	<0.001	<0.001
FE^5^	9.80 ± 2.85^b^	40.5 ± 1.5^b^	79.2 ± 12.0^a^	76.9 ± 16.4^a^	83.0 ± 5.39^a^	80.0 ± 11.5^a^	<0.001	<0.001	0.001
PER^6^	0.19 ± 0.06^c^	0.79 ± 0.03^b^	1.55 ± 0.24^a^	1.51 ± 0.32^a^	1.63 ± 0.11^a^	1.57 ± 0.22^a^	<0.001	<0.001	<0.001
PRE^7^	7.35 ± 0.47^b^	27.2 ± 3.64^a^	25.5 ± 1.56^a^	26.4 ± 1.82^a^	30.9 ± 4.70^a^	27.0 ± 1.09^a^	<0.001	<0.001	<0.001
FI^8^	34.4 ± 5.40	40.6 ± 2.67	37.4 ± 0.99	37.7 ± 1.63	38.2 ± 2.47	37.9 ± 0.96	0.251	0.427	0.254
Survival (%)	66.7 ± 18.0^b^	93.3 ± 6.11^a^	100 ± 0.00^a^	100 ± 0.00^a^	96.0 ± 4.00^a^	97.3 ± 2.31^a^	0.001	0.002	0.001

The experimental diets were formulated to contain valine by 4, 8, 12, 16, 20, and 24 g/kg diet. Values are means from triplicate groups of fish where the values in each row with the different superscripts are significantly different (*P* < 0.05).  ^*∗*^Significance probability associate with *F*-statistic. ^1^Final body weight (g). ^2^Weight gain (%) = (final body weight − initial body weight)/initial body weight × 100. ^3^Specific growth rate (%/d) = (log_e_ final weight − log_e_ initial weight)/feeding days × 100. ^4^Feed conversion ratio = dry feed fed/wet weight gain. ^5^Feed efficiency (%) = wet weight gain/dry feed fed × 100. ^6^Protein efficiency ratio = wet weight gain/total protein given. ^7^Protein retention efficiency (%) = protein gain/total protein given × 100. ^8^Feed intake (g/fish) = dry feed consumed (g)/fish.

**Table 5:** Biological assessment of digestive organs and condition factor of juvenile olive flounder (*P. olivaceus*) fed the experimental diets for 13 weeks.

	Dietary valine levels (g/kg)						Pr > *F* ^*∗*^		
	4	8	12	16	20	24	ANOVA	Linear	Quadratic
HSI^1^	0.81 ± 0.17^c^	0.92 ± 0.19^bc^	1.31 ± 0.38^a^	1.04 ± 0.28^abc^	1.11 ± 0.24^abc^	1.12 ± 0.13^abc^	0.002	0.009	0.020
SSI^2^	1.60 ± 0.15	1.60 ± 0.17	1.90 ± 0.27	1.84 ± 0.38	1.77 ± 0.15	1.87 ± 0.31	0.055	0.057	0.232
ISI^3^	1.90 ± 0.37^a^	1.94 ± 0.34^a^	1.74 ± 0.26^ab^	1.79 ± 0.17^ab^	1.48 ± 0.14^b^	1.45 ± 0.23^b^	<0.001	<0.001	0.329
CF^4^	0.60 ± 0.07^c^	0.69 ± 0.05^bc^	0.78 ± 0.08^ab^	0.79 ± 0.03^ab^	0.86 ± 0.08^a^	0.82 ± 0.10^a^	<0.001	<0.001	0.001

The experimental diets were formulated to contain valine by 4, 8, 12, 16, 20, and 24 g/kg diet. Values are means from triplicate groups of fish where the values in each row with the different superscripts are significantly different (*P*  < 0.05).  ^*∗*^Significance probability associate with *F*-statistic. ^1^Hepatosomatic index = (liver weight × 100)/fish body weight. ^2^Stomachsomatic index = (stomach weight × 100)/fish body weight. ^3^Intestinesomatic index = (intestine weight × 100)/fish body weight. ^4^Condition Factor = (fish body weight / fish body length^3^) × 100.

**Table 6:** Whole-body proximate composition (% of wet basis) of juvenile olive flounder (*P. olivaceus*) fed the experimental diets for 13 weeks.

Item	Dietary valine levels (g/kg)						Pr > *F* ^*∗*^		
	4	8	12	16	20	24	ANOVA	Linear	Quadratic
Protein	19.1 ± 0.15^b^	23.1 ± 0.70^a^	22.3 ± 0.42^a^	22.5 ± 0.21^a^	23.4 ± 0.51^a^	22.7 ± 0.25^a^	<0.001	<0.001	<0.001
Lipid	2.41 ± 0.07	2.66 ± 0.18	2.56 ± 0.21	2.69 ± 0.04	2.71 ± 0.27	2.67 ± 0.24	0.430	0.110	0.398
Ash	3.08 ± 0.25^b^	3.68 ± 0.18^a^	3.79 ± 0.16^a^	3.65 ± 0.05^a^	3.65 ± 0.05^a^	3.60 ± 0.29^a^	0.048	0.077	0.023
Moisture	74.6 ± 0.66^a^	70.6 ± 0.36^b^	70.8 ± 0.04^b^	70.3 ± 0.76^b^	69.7 ± 0.61^b^	70.2 ± 0.40^b^	0.001	<0.001	0.001

The experimental diets were formulated to contain valine by 4, 8, 12, 16, 20, and 24 g/kg diet. Values are means from triplicate groups of fish where the values in each row with the different superscripts are significantly different (*P*  < 0.05).  ^*∗*^Significance probability associate with the *F*-statistic.

**Table 7:** Amino acid profiles (% of protein) in the whole body of juvenile olive flounder (*P. olivaceus*) fed the experimental diets for 13 weeks.

AAs	Dietary valine levels (g/kg)						Pr > *F* ^*∗*^		
	4	8	12	16	20	24	ANOVA	Linear	Quadratic
*Essenial amino acids*									
Valine	4.67 ± 0.07^b^	5.16 ± 0.04^ab^	5.24 ± 0.22^a^	5.26 ± 0.20^a^	5.34 ± 0.20^a^	5.30 ± 0.09^a^	0.033	0.006	0.037
Histidine	2.91 ± 0.13	2.94 ± 0.39	2.76 ± 0.30	2.56 ± 0.02	2.54 ± 0.01	2.54 ± 0.09	0.271	0.056	0.738
Isoleucine	3.84 ± 0.24^b^	4.59 ± 0.15^ab^	4.48 ± 0.03^ab^	4.66 ± 0.18^ab^	4.76 ± 0.26^a^	4.85 ± 0.37^a^	0.037	0.005	0.144
Leucine	6.53 ± 0.25^b^	7.31 ± 0.42^ab^	7.44 ± 0.06^a^	7.64 ± 0.29^a^	7.60 ± 0.12^a^	7.74 ± 0.14^a^	0.021	0.003	0.051
Lysine	8.17 ± 0.51	9.27 ± 0.58	9.27 ± 0.01	8.36 ± 0.11	8.55 ± 0.11	8.57 ± 0.01	0.055	0.598	0.066
Phenylalanine	3.81 ± 0.04	4.11 ± 0.07	3.97 ± 0.13	4.09 ± 0.02	4.14 ± 0.03	4.09 ± 0.14	0.057	0.062	0.130
Threonine	3.89 ± 0.25^b^	4.35 ± 0.03^a^	4.37 ± 0.04^a^	4.34 ± 0.09^a^	4.39 ± 0.06^a^	4.58 ± 0.11^a^	0.016	0.002	0.170
Arginine	7.59 ± 0.49	7.27 ± 0.13	7.31 ± 0.10	7.42 ± 0.19	7.43 ± 0.04	7.36 ± 0.15	0.794	0.707	0.482
*Nonessential amino acids*									
Alanine	7.64 ± 0.29	7.02 ± 0.13	7.31 ± 0.01	7.42 ± 0.14	7.39 ± 0.10	7.36 ± 0.19	0.116	0.882	0.181
Aspartic acid	10.1 ± 0.46	10.5 ± 0.08	10.2 ± 0.11	10.6 ± 0.23	10.6 ± 0.20	10.5 ± 0.04	0.380	0.132	0.433
Glycine	10.9 ± 1.29	8.74 ± 0.80	8.98 ± 0.42	9.34 ± 0.10	9.04 ± 0.12	8.77 ± 0.12	0.096	0.051	0.132
Glutamic acid	14.4 ± 0.52	16.3 ± 0.67	16.2 ± 1.00	15.7 ± 0.09	15.9 ± 0.17	16.2 ± 0.32	0.088	0.070	0.114
Proline	6.10 ± 0.67	5.85 ± 0.89	5.86 ± 0.24	5.92 ± 0.11	5.88 ± 0.15	5.84 ± 0.55	0.995	0.717	0.811
Serine	4.31 ± 0.02^bc^	4.24 ± 0.03^c^	4.38 ± 0.06^abc^	4.49 ± 0.03^ab^	4.42 ± 0.06^ab^	4.51 ± 0.07^a^	0.010	0.001	0.993
Tyrosine	2.46 ± 0.30	2.58 ± 0.13	2.69 ± 0.11	2.67 ± 0.02	2.65 ± 0.07	2.83 ± 0.04	0.337	0.055	0.834

The experimental diets were formulated to contain valine by 4, 8, 12, 16, 20, and 24 g/kg diet. Values are means from triplicate groups of fish where the values in each row with the different superscripts are significantly different (*P*  < 0.05).  ^*∗*^Significance probability associate with *F*-statistic.

**Table 8:** Hematological parameters of juvenile olive flounder (*P. olivaceus*) fed the experimental diets for 13 weeks.

Item	Dietary valine levels (g/kg)						Pr > *F* ^*∗*^		
	4	8	12	16	20	24	ANOVA	Linear	Quadratic
Cholesterol (mg/dL)	33.4 ± 3.61^c^	103 ± 4.85^b^	115 ± 3.89^b^	102 ± 9.89^b^	109 ± 5.20^b^	142 ± 8.96^a^	<0.001	<0.001	<0.001
Hematocrit (%)	23.0 ± 2.63	21.0 ± 2.64	22.4 ± 1.82	21.0 ± 2.34	24.0 ± 0.75	21.0 ± 2.00	0.062	0.817	0.340
Total protein (g/dL)	1.91 ± 0.31^b^	2.66 ± 0.22^a^	2.66 ± 0.35^a^	2.52 ± 0.09^a^	2.87 ± 0.27^a^	2.72 ± 0.06^a^	<0.001	<0.001	0.012
Glucose (mg/dL)	33.7 ± 5.53^a^	27.7 ± 5.44^ab^	25.2 ± 4.85^ab^	19.7 ± 5.97^b^	21.3 ± 3.92^b^	22.0 ± 4.01^b^	0.006	0.001	0.029
AST^1^ (U/L)	36.2 ± 3.54^a^	24.1 ± 3.41^b^	19.9 ± 2.27^b^	21.9 ± 2.79^b^	10.5 ± 1.78^c^	11.5 ± 2.72^c^	<0.001	<0.001	0.012
ALT^2^ (U/L)	10.3 ± 3.21	8.75 ± 0.91	8.40 ± 1.48	8.40 ± 1.64	5.76 ± 1.76	5.92 ± 0.39	0.059	0.006	0.962

The experimental diets were formulated to contain valine by 4, 8, 12, 16, 20, and 24 g/kg diet. Values are means from triplicate groups of fish where the values in each row with the different superscripts are significantly different (*P*  < 0.05).  ^*∗*^Significance probability associate with *F*-statistic. ^1^Aspartate aminotransferase. ^2^Alanine aminotransferase.

**Table 9:** Nonspecific immune responses and antioxidant capacity of juvenile olive flounder (*P. olivaceus*) fed the experimental diets for 13 weeks.

Item	Dietary valine levels (g/kg)						Pr > *F* ^*∗*^		
	4	8	12	16	20	24	ANOVA	Linear	Quadratic
NBT^1^	0.87 ± 0.27^b^	1.26 ± 0.24^a^	1.55 ± 0.08^a^	1.51 ± 0.15^a^	1.37 ± 0.15^a^	1.49 ± 0.11^a^	<0.001	<0.001	<0.001
MPO^2^	0.13 ± 0.01^b^	0.16 ± 0.03^ab^	0.17 ± 0.02^ab^	0.19 ± 0.02^a^	0.15 ± 0.03^ab^	0.18 ± 0.04^a^	0.015	0.018	0.097
Lysozyme^3^	13.2 ± 1.01	11.0 ± 0.87	18.4 ± 2.66	16.8 ± 3.90	12.1 ± 2.29	17.2 ± 4.19	0.054	0.147	0.477
Ig^4^	26.4 ± 7.82^b^	37.8 ± 7.83^ab^	33.3 ± 7.30^ab^	31.1 ± 9.48^ab^	38.5 ± 12.0^ab^	45.6 ± 6.27^a^	0.037	0.008	0.473
SOD^5^	67.2 ± 4.21^b^	80.9 ± 6.32^a^	80.7 ± 6.37^a^	82.8 ± 8.55^a^	77.1 ± 8.66^ab^	79.6 ± 7.31^a^	<0.001	0.009	0.001
GPx^6^	65.6 ± 9.26^b^	77.2 ± 9.90^ab^	81.4 ± 6.61^a^	87.7 ± 9.49^a^	90.6 ± 7.65^a^	77.0 ± 7.56^ab^	<0.001	0.001	<0.001
Antiprotease^7^	23.0 ± 2.15	28.4 ± 2.65	27.8 ± 1.63	27.9 ± 2.23	28.1 ± 0.34	27.1 ± 2.82	0.408	0.253	0.102

The experimental diets were formulated to contain valine by 4, 8, 12, 16, 20, and 24 g/kg diet. Values are means from triplicate groups of fish where the values in each row with the different superscripts are significantly different (*P*  < 0.05).  ^*∗*^Significance probability associate with *F*-statistic. ^1^NBT, nitro blue tetrazolium (absorbance). ^2^MPO, myeloperoxidase (*µ*g/mL). ^3^Lysozyme (*µ*g/mL). ^4^Ig, immunoglobulin (mg/mL). ^5^SOD, superoxide dismutase (% inhibition). ^6^GPx, glutathione peroxidase (mU/mL). ^7^Antiprotease (% inhibition).

**Table 10:** Intestine histology of juvenile olive flounder (*P. olivaceus*) fed the experimental diets for diets for 13 weeks.

Item	Dietary valine levels (g/kg)						Pr > *F* ^*∗*^		
	4	8	12	16	20	24	ANOVA	Linear	Quadratic
Mucosal fold height (*μ*m)	472 ± 186^c^	600 ± 155^b^	723 ± 230^a^	759 ± 235^a^	764 ± 191^a^	786 ± 195^a^	<0.001	<0.001	0.003
Mucosal fold width (*μ*m)	101 ± 17.9	106 ± 17.2	112 ± 32.7	113 ± 22.5	118 ± 15.9	106 ± 19.0	0.651	0.251	0.350
Lamina propria thickness (*μ*m)	62.0 ± 20.2	63.2 ± 8.47	62.6 ± 14.7	60.6 ± 12.0	60.7 ± 9.18	53.3 ± 13.2	0.280	0.076	0.120
Submucosa thickness (*μ*m)	138 ± 27.7^c^	182 ± 23.9^bc^	202 ± 24.2^b^	201 ± 37.3^b^	250 ± 39.4^a^	262 ± 56.1^a^	<0.001	<0.001	0.378
Muscularis thickness (*μ*m)	190 ± 48.2^d^	261 ± 25.1^c^	304 ± 28.5^bc^	342 ± 44.7^ab^	323 ± 40.7^ab^	376 ± 65.7^a^	<0.001	<0.001	0.057

The experimental diets were formulated to contain valine by 4, 8, 12, 16, 20, and 24 g/kg diet. Values are means from triplicate groups of fish where the values in each row with the different superscripts are significantly different (*P*  < 0.05).  ^*∗*^Significance probability associate with the *F*-statistic.

## Data Availability

Data will be available on request.
